# PfEMP1 A-Type ICAM-1-Binding Domains Are Not Associated with Cerebral Malaria in Beninese Children

**DOI:** 10.1128/mBio.02103-20

**Published:** 2020-11-17

**Authors:** V. Joste, E. Guillochon, J. Fraering, B. Vianou, L. Watier, S. Jafari-Guemouri, M. Cot, S. Houzé, A. Aubouy, J. F. Faucher, N. Argy, G. I. Bertin

**Affiliations:** a Université de Paris, MERIT, IRD, Paris, France; b Institut de Recherche Clinique du Bénin (IRCB), Cotonou, Bénin; c Department of Biostatistics, Biomathematics, Pharmacoepidemiology and Infectious Diseases (B2PHI), Inserm, UVSQ, Institut Pasteur, Université Paris-Saclay, Paris, France; d Parasitology Laboratory, Bichat-Claude Bernard hospital, Paris, France; e Malaria National Reference Center, Bichat-Claude Bernard hospital, Paris, France; f Université de Toulouse, PHARMADEV, IRD, UPS, Toulouse, France; g Université de Limoges, NET, INSERM, Limoges, France; Johns Hopkins Bloomberg School of Public Health; University of Pittsburgh

**Keywords:** cerebral malaria, *var* genes, cytoadherence, dual receptor binding, ICAM-1-binding motif

## Abstract

PfEMP1 is the major antigen involved in Plasmodium falciparum-infected erythrocyte sequestration in cerebrovascular endothelium. While some PfEMP1 domains have been associated with clinical phenotypes of malaria, formal associations between the expression of a specific domain and the adhesion properties of clinical isolates are limited. In this context, 73 cerebral malaria (CM) and 98 uncomplicated malaria (UM) Beninese children were recruited. We attempted to correlate the cytoadherence phenotype of Plasmodium falciparum isolates with the clinical presentation and the expression of specific PfEMP1 domains. Cytoadherence level on Hbec-5i and CHO-ICAM-1 cell lines and *var* genes expression were measured. We also investigated the prevalence of the ICAM-1-binding amino acid motif and dual receptor-binding domains, described as a potential determinant of cerebral malaria pathophysiology. We finally evaluated IgG levels against PfEMP1 recombinant domains (CIDRα1.4, DBLβ3, and CIDRα1.4-DBLβ3). CM isolates displayed higher cytoadherence levels on both cell lines, and we found a correlation between CIDRα1.4-DBLβ1/3 domain expression and CHO-ICAM-1 cytoadherence level. Endothelial protein C receptor (EPCR)-binding domains were overexpressed in CM isolates compared to UM whereas no difference was found in ICAM-1-binding DBLβ1/3 domain expression. Surprisingly, both CM and UM isolates expressed ICAM-1-binding motif and dual receptor-binding domains. There was no difference in IgG response against DBLβ3 between CM and UM isolates expressing ICAM-1-binding DBLβ1/3 domain. It raises questions about the role of this motif in CM pathophysiology, and further studies are needed, especially on the role of DBLβ1/3 without the ICAM-1-binding motif.

## Abstract (precis)

Cerebral malaria pathophysiology remains unknown despite extensive research. PfEMP1 proteins have been identified as the main *Plasmodium* antigen involved in cerebrovascular endothelium sequestration, but it is unclear which *var* gene domain is involved in *Plasmodium* cytoadhesion. EPCR binding is a major determinant of cerebral malaria whereas the ICAM-1-binding role is still questioned. Our study confirmed the EPCR-binding role in CM pathophysiology with a major overexpression of EPCR-binding domains in CM isolates. In contrast, ICAM-1-binding involvement appears less obvious with A-type ICAM-1-binding and dual receptor-binding domain expression in both CM and UM isolates. We did not find any variations in ICAM-1-binding motif sequences in CM compared to UM isolates. UM and CM patients infected with isolates expressing the ICAM-1-binding motif displayed similar IgG levels against DBLβ3 recombinant protein. Our study raises interrogations about the role of these domains in CM physiopathology and questions their use in vaccine strategies against cerebral malaria.

## INTRODUCTION

Cerebral malaria (CM) is the most severe form of Plasmodium falciparum (P. falciparum) infection, mainly affecting children under 5 years in areas of endemicity (1). One of the most-described pathophysiologic mechanisms of CM is the ability of P. falciparum-infected erythrocytes (iE) to adhere to the cerebrovascular endothelium through variant surface antigen (VSA) proteins. These proteins are expressed on the iE cell membrane and bind to multiple endothelial receptors (2). In CM, this phenomenon causes brain swelling, coma, and even death (3). Currently, there is no specific marker for CM, but histopathology of malarial retinopathy (MR) (4, 5) correlates with cerebral pathology.

The major VSA involved in iE sequestration is Plasmodium falciparum erythrocyte membrane protein 1 (PfEMP1). This parasitic protein is encoded by *var* genes (6), a hypervariable multigenic family classified in four groups, A, B, C, and E, and two intermediate groups, B/A and B/C (7). PfEMP1 proteins from A (8, 9) and B/A (10) groups are associated with severe malaria (SM). PfEMP1 sequences are divided into two types of hypervariable extracellular domains: cysteine-rich interdomain regions (CIDR) and Duffy-binding-like (DBL). They have been classified into domain cassettes (DCs), composed of at least two conserved domains, DBL/CIDR (11).

Several studies on laboratory P. falciparum strains selected to adhere to brain microvascular endothelial cells have revealed preferential expression of certain PfEMP1 DCs such as DC8 (DBLα2-CIDRα1.1/8-DBLβ12-DBLγ4/6) and DC13 (DBLα1.7-CIDRα1.4) (12, 13). These DCs are predicted to bind to endothelial protein C receptor (EPCR) (15) through CIDRα1 domains (16) and are overexpressed in CM clinical isolates (10, 17–19). Importantly, inhibition of EPCR binding did not block the adherence of iE expressing DC8 and DC13 to brain endothelial cells, and EPCR binding alone is not sufficient to fully explain cytoadhesion level (20). These elements suggest the implication of another receptor in the cytoadherence mechanism.

Intercellular adhesion molecule 1 (ICAM-1) receptor has previously been proposed to be implicated in iE adhesion, and ICAM-1 overexpression in cerebrovascular tissue has been shown (21). Moreover, it has been demonstrated that EPCR and ICAM-1 are coinvolved in iE binding to brain cells (22). In this context, an ICAM-1-binding amino acid sequence, found in A-type DBLβ1/3 domains and mainly associated with CM, was recently identified (23–25). Interestingly, each DBLβ1/3 domain predicted to bind ICAM-1 is preceded by an EPCR-binding CIDRα1 domain which defines a dual receptor-binding PfEMP1 sequence, associated with CM (23). Jensen et al. proposed a pathogenic cascade where the iE first binds to EPCR, leading to a proinflammatory cytokine release and increased expression of ICAM-1 (26).

Despite extensive research, few formal associations between the expression of specific domains of PfEMP1 and the adhesion properties of clinical isolates of P. falciparum have been demonstrated (18, 19, 27). In order to fill this gap, we conducted a comparative study on CM and uncomplicated malaria (UM) in Beninese children, in which we attempted to correlate the cytoadherence phenotype of P. falciparum isolates with clinical presentation and the expression of specific domains of PfEMP1. We also focused on the ICAM-1-binding motif and its implication in CM pathogenesis.

## RESULTS

### Recruitment and sample description.

A total of 171 patients were included, comprising 73 CM and 98 UM cases. Two UM cases were coinfected with Plasmodium malariae and subsequently excluded from downstream analysis. Clinical and biological characteristics of recruited patients are described in Table 1. Compared to UM, CM patients presented a lower age and blood pressure and a higher pulse and respiratory rate (*P *< 0.0001). Moreover, CM patients had more frequent hepatomegaly (*P* < 0.0001) and splenomegaly (*P* = 0.0046). Sixty-seven percent of diagnosed CM cases presented retinopathy. CM cases had lower hemoglobin and blood glucose concentrations, lower platelet counts, higher creatinine and bilirubin concentrations, and higher leukocyte counts (*P* < 0.0001). Notably, 35% of children with CM died in our study in spite of adequate management including parenteral treatment with artesunate. There was no difference in multiplicity of infection (MOI) between CM and UM isolates (4 [3 to 9] versus 4 [3 to 7]). Values in parentheses and brackets are median [10-90th percentile].

**TABLE 1:** Clinical characteristics of the patients included during the study^*a*^

	Cerebral malaria (*n* = 73)	Uncomplicated malaria (*n* = 96)	*P* value
Age (mo)	43.4 (29.9–63.2)	59.9 (36–70)	<0.0001
Gender, no. female/total no. (% female)	44/73 (60%)	42/96 (44%)	0.033
Pulse rate (beats/min)	152 (122.2–178)	120 (100–160)	<0.0001
Respiratory rate (beats/min)	48 (34.4–65.6)	40 (36–48)	<0.0001
Systolic blood pressure (mm Hg)	90 (70–100)	110 (100–120)	<0.0001
Parasitemia (parasites/μl)	60,900 (283–763,040)	39,180 (3,784–253,360)	
Hemoglobinemia (g/dl)	5.4 (3.3–8.7)	9.4 (7–11.3)	<0.0001
Leukocytes (G/liter)	12.8 (7.4–29.4)	7.2 (4.6–10.4)	<0.0001
Neutrophils (G/liter)	8.1 (3.9–17.9)	3.3 (1.8–6.6)	<0.0001
Lymphocytes (G/liter)	4.3 (1.7–11.1)	2.2 (1.1–4.1)	<0.0001
Platelets (G/liter)	85 (42.3–214.2)	177 (79.5–346.5)	<0.0001
Creatinine (mg/liter)	5 (2.8–8.2)	3 (3–4.3)	<0.0001
Total bilirubin (mg/liter)	26 (9.8–70.2)	9.3 (4.7–20.9)	<0.0001
Conjugated bilirubin (mg/liter)	13.4 (3.4–30.4)	4.3 (2–9.3)	<0.0001
Blood glucose (g/liter)	0.8 (0.1–1.3)	1 (0.8–1.3)	<0.0001
Urea (g/liter)	0.15 (0.1–0.37)	NA	
Lactates (mmol/liter)	6.2 (2.4–9.9)	NA	
Albuminemia (g/liter)	27 (21–33)	NA	
GPT (UI/liter)^*b*^	38 (14–114)	NA	
Bicarbonates (mmol/liter)	14.8 (6–23.9)	NA	
Hepatomegaly (positive %)	53.4	9.5	<0.0001
Splenomegaly (positive %)	37	18.1	0.0046
Malarial retinopathy		NA	
Yes	35 (48)		
No	17 (23)		
Not evaluated	21 (29)		
Blantyre score			
0	1 (1.4)	NA	
1	22 (30.1)	NA	
2	50 (68.5)	NA	
3	0	NA	
4	0	NA	
5	0	NA	
Mortality (%)	34.8%	NA	

a Nonparametric results were represented as median (10th to 90th percentile) and proportions as *n* (%). Statistical differences between UM and CM were calculated using the Mann-Whitney U-test or the χ^2^ test. Only significative *P* values of <0.05 were indicated. NA, nonattributable.

b Glutamate-pyruvate transaminase.

### Cytoadherence of isolates on Hbec-5i and CHO-ICAM-1 cell lines.

**FIG 1:**
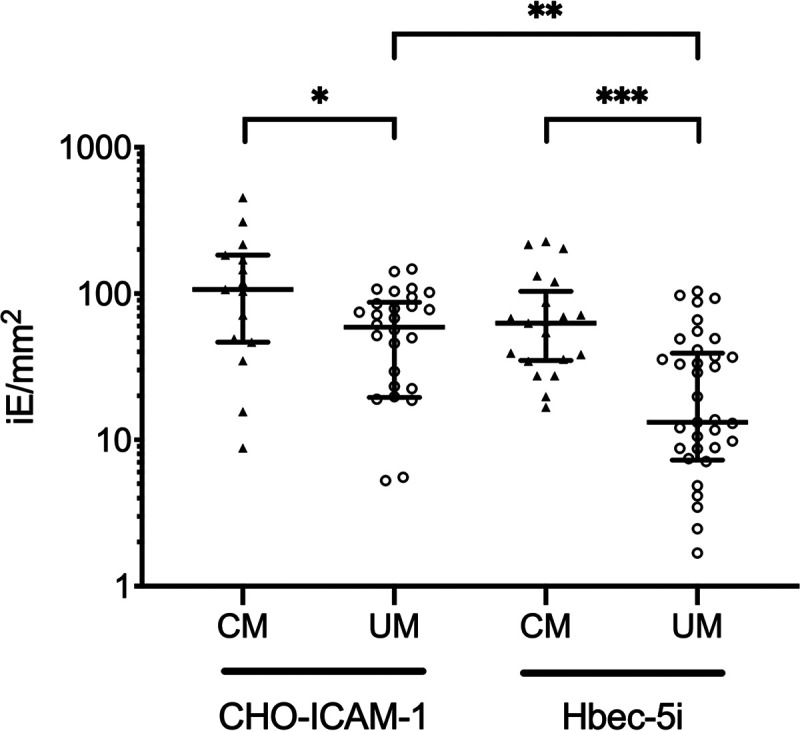
Cytoadherence of UM and CM isolates. Median and interquartile range of bound infected erythrocytes (iE) are indicated. Hbec-5i cytoadherence was evaluated for 21 CM and 35 UM isolates. CHO-ICAM-1 cytoadherence was evaluated for 15 CM and 28 UM isolates. *P* values were calculated with Mann-Whitney U-test. *, *P* < 0.05; **, *P* < 0.01; ***, *P* < 0.0001; NS, nonsignificant.

On Hbec-5i, the cytoadherence value was 63 (27 to 203) iE/mm^2^ for CM isolates (*n* = 21) and 13 (7 to 62) iE/mm^2^ for UM isolates (*n* = 35) (*P* < 0.0001). The cytoadherence assay was also performed on CHO-ICAM-1, for which the cytoadherence value was 107 (44 to 273) iE/mm^2^ for CM isolates (*n* = 15) and 59 (4 to 108) iE/mm^2^ for UM isolates (*n* = 28) (*P* = 0.01) (Fig. 1). In addition, cytoadherence levels on Hbec-5i and CHO-ICAM-1 cells were similar in CM isolates, while cytoadherence to CHO-ICAM-1 was higher in UM isolates (*P* = 0.0005). We performed cytoadherence assays with the HB3 strain selected on Hbec-5i or CHO-ICAM-1 cell lines. HB3-Hbec-5i and HB3-ICAM-1 displayed 22- and 7-times-higher cytoadherence values, respectively, than the median cytoadherence value of isolates. We also compared HB3-ICAM-1 cytoadherence values on both CHO-ICAM-1 and the parental CHO cell lines and demonstrated that ICAM-1 receptor explained at least 75% of cytoadherence on CHO-ICAM-1 cell lines.

### *var* gene expression quantification.

Our primer set displayed good coverage on the CIVIC and Pf3K data sets (see Table S1a and b in the supplemental material). All CM and UM isolates were typed for *var* gene expression (Table 2). CM isolates overexpressed EPCR-binding domains (CIDRα1.1, CIDRα1.4 to 1.8), ICAM-1-binding domain (DBLβ5), and double domains (CIDRα1.4-DBLβ1/3, CIDRα1.6-DBLβ1/3, CIDRα1.7-DBLβ1/3) as well as domains without predicted binding characteristics (CIDRα1.2, DBLξ3, DBLε2, DBLγ1) compared to UM isolates. No difference was observed for CD36-binding domains (CIDRα2.3/5/6/7/9/10), CIDRδ- and ICAM-1-binding DBLβ1/3 domain. Primers targeting DBLα2/1.1/2/4/7 domains, predicted to precede EPCR-binding CIDRα1, reported a higher level in CM isolates (*P* < 0.0001). We did not observe any difference in *var* genes domain expression between positive and negative MR patients (Table S2).

**TABLE 2:** Relative quantification of *var* gene transcript levels^*a*^

Domain	Group	Predicted receptor	CM		UM		*P* value
			*n*	Median (10th–90th percentile)	*n*	Median (10th–90th percentile)	
CIDRα1.1	B/A	EPCR	68	0.056 (0–1.16)	96	0.0041 (0–0.53)	0.0052
CIDRα1.8	B/A	EPCR	70	0.00071 (0–0.075)	95	0 (0–0.018)	0.0029
CIDRα1.4	A	EPCR	70	0.17 (0–3.7)	96	0.012 (0–1.1)	0.0020
CIDRα1.5	A	EPCR	70	0.0057 (4.4 × 10^−5^–0.071)	95	0.00063 (0 to 0.014)	<0.0001
CIDRα1.6	A	EPCR	69	0.01 (0–0.13)	96	0.0020 (0–0.017)	0.00027
CIDRα1.7	A	EPCR	69	0.0057 (0.00015–0.057)	85	0.001 (2.3 × 10^−5^–0.0094)	<0.0001
CIDRα1.2			69	0.0072 (0–0.036)	96	0.0033 (0–0.013)	0.010
CIDRδ	A		69	0.0088 (0–0.42)	96	0.0032 (0–0.12)	
CIDRα2.3/5/6/7/9/10	B	CD36	70	0.00063 (0–0.0069)	95	0.00026 (0–0.0043)	
DBLα1.7	A		66	0.14 (0–8.6)	92	0.12 (0–4.94)	
DBLα2/1.1/2/4/7	A		70	0.43 (0.077–2.35)	95	0.15 (0.030–0.61)	<0.0001
DBLβ1/3-motif	A	ICAM-1	68	6.9 × 10^−5^ (0–0.19)	93	0 (0–0.088)	
DBLβ5	B	ICAM-1	69	0.022 (0–0.19)	96	0.0026 (0–0.11)	0.00062
DBLε2			69	0 (0–0.0034)	96	0 (0–0.0034)	0.011
DBLξ3			70	0.0025 (0–0.019)	96	0 (0–0.009)	<0.0001
DBLγ1			70	0.0016 (0.0002–0.0081)	93	0.00042 (0–0.0032)	0.001
CIDRα1.4-DBLβ1/3	A	EPCR ± ICAM-1	70	0.038 (0–0.31)	92	0.0046 (0–0.062)	<0.0001
CIDRα1.6-DBLβ1/3	A	EPCR ± ICAM-1	70	0.017 (0–0.12)	95	0.0022 (0–0.040)	<0.0001
CIDRα1.7-DBLβ1/3	A	EPCR ± ICAM-1	66	0.065 (0.00043–0.58)	90	0.0033 (0–0.059)	<0.0001

a Results are expressed after normalization with P90 quantification. Nonparametric results are presented as median (10th to 90th percentile). Results were considered significative when *P* value was <0.05. *P* values were calculated with Mann-Whitney U-test. Only significant *P* values of <0.05 are indicated. Number of isolates (*n*) in the analysis is specified.

10.1128/mBio.02103-20.6 TABLE S1 (a) *In silico* newly designed RT-qPCR *var* gene primers based on CIVIC data set. Coverage and specificity values were calculated on CIVIC and Pf3K (African countries) data sets. (b) *In silico* designed primers used in Sanger sequencing. Download Table S1, PDF file, 0.6 MB. Copyright © 2020 Joste et al. 2020 Joste et al. This content is distributed under the terms of the Creative Commons Attribution 4.0 International license.

10.1128/mBio.02103-20.7 TABLE S2 Transcript levels of *var* genes and cytoadherence value between malarial retinopathy and normal fundus isolates. Results are expressed after normalization with P90 quantification. Results shown are median with 10th and 90th percentiles. *P* values were calculated with Mann-Whitney U-test. Number of isolates (*n*) in the analysis is specified. None of the median comparisons was statistically significant. Download Table S2, DOCX file, 0.01 MB. Copyright © 2020 Joste et al. 2020 Joste et al. This content is distributed under the terms of the Creative Commons Attribution 4.0 International license.

**FIG 2:**
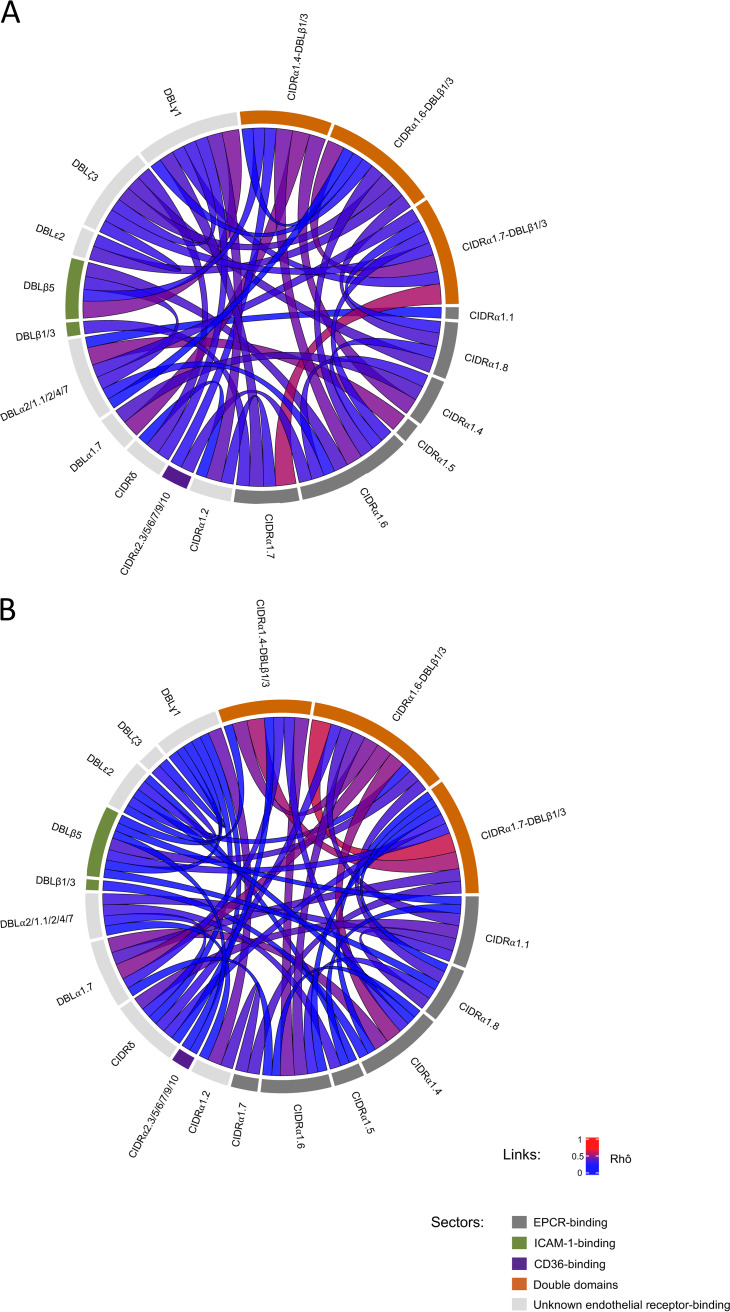
Chord diagram. Correlations between *var* genes expressions were calculated with Spearman rank test for CM isolates (A) and UM isolates (B). Only correlations with *P* value adjusted with Benjamini-Hochberg correction <0.1 are represented.

Associations between *var* genes expression were also evaluated. Expected connections (such as CIDRα1.7 and CIDRα1.7-DBLβ1/3) were observed. Interestingly, a significant correlation was shown between DBLξ3 expression and double domains in CM isolates (Fig. 2 and Table S3).

10.1128/mBio.02103-20.8 TABLE S3 (a) Spearman’s rank correlation coefficient rho for UM patients. *P* values were adjusted with Benjamini-Hochberg correction. (b) Spearman’s rank correlation coefficient rho for cytoadherence of UM isolates. *P* values were adjusted with Benjamini-Hochberg correction. (c) Spearman’s rank correlation coefficient rho for CM patients. *P* values were adjusted with Benjamini-Hochberg correction. (d) Spearman’s rank correlation coefficient rho for cytoadherence of CM isolates. *P* values were adjusted with Benjamini-Hochberg correction. Download Table S3, XLSX file, 0.04 MB. Copyright © 2020 Joste et al. 2020 Joste et al. This content is distributed under the terms of the Creative Commons Attribution 4.0 International license.

### Partial correlation between cytoadherence and *var* gene expression.

Since expression of no more than one *var* gene was associated with Hbec-5i cytoadherence (Table S3), partial correlation only was studied for CHO-ICAM-1. Expression levels of CIDRα1.4 and DBLβ1/3 in CM isolates and CIDRα1.4-DBLβ1/3 and CIDRα1.6 in UM isolates were associated independently with CHO-ICAM-1 cytoadherence. Partial-correlation results are summarized in Table S4.

10.1128/mBio.02103-20.9 TABLE S4 Spearman partial correlation. As no univariate correlation was significative for the CM population and only one was significant for the UM population, partial correlation was not calculated for Hbec-5i cytoadherence. Download Table S4, DOCX file, 0.01 MB. Copyright © 2020 Joste et al. 2020 Joste et al. This content is distributed under the terms of the Creative Commons Attribution 4.0 International license.

### ICAM-1-binding motif sequencing.

Sanger sequencing was performed on isolates with ICAM-1-binding DBLβ1/3 domain expression in reverse transcription-quantitative PCR (RT-qPCR) (34 CM and 38 UM). We obtained 32 DBLβ1/3 sequences (17 CM and 15 UM) with an ICAM-1-binding motif (Table S5). We successfully sequenced 11 CIDRα-ICAM-1-binding DBLβ1/3 dual domains and found that all CIDRα domains were predicted to bind EPCR. A phylogenetic analysis showed no clear association between clinical groups and ICAM-1-binding motif sequence (data not shown).

10.1128/mBio.02103-20.10 TABLE S5 ICAM-1-binding motif sequence. Homology sequence percentages are calculated compared to PFD1235W. Download Table S5, XLSX file, 0.01 MB. Copyright © 2020 Joste et al. 2020 Joste et al. This content is distributed under the terms of the Creative Commons Attribution 4.0 International license.

### Recombinant protein sequences and binding property validations.

PfEMP1 recombinant domains purity was evaluated by SDS-PAGE and Coomassie blue staining (Fig. S1a) and the domains’ sizes were as expected (35 kDa for CIDRα1.4, 65 kDa for DBLβ3, and 100 kDa for the CIDRα1.4-DBLβ3 double domain). To ensure the amino acid sequences of our recombinant proteins, a mass spectrometry analysis was carried out (Text S1 and Fig. S1b). All identified peptides matched and covered 70% of the sequence. Furthermore, for the CIDRα1.4-DBLβ3 domain, a single peptide overlapping both CIDRα1.4 and DBLβ3 domains was detected (blue highlighting). We validated the interactions between the recombinant domains and the receptors by ligand receptor assay (29) and far-Western blotting (30) (see Text S1 and Fig. S2 and S3).

10.1128/mBio.02103-20.1 TEXT S1 (1.) Digestion of recombinant PfEMP1 domains produced. (2.) Mass spectrometry analysis of recombinant PfEMP1 domains produced. (3.) Assessment of recombinant domain binding activity on EPCR and ICAM-1 receptor. Download Text S1, DOCX file, 0.02 MB. Copyright © 2020 Joste et al. 2020 Joste et al. This content is distributed under the terms of the Creative Commons Attribution 4.0 International license.

10.1128/mBio.02103-20.2 FIG S1a SDS-PAGE and Coomassie blue staining of different recombinant PfEMP1 domain purifications. (A) Purification of CIDRα1.4-DBLβ3 (30°C, 4-h induction) produced in Schuffle cells. (B) Purification of CIDRα1.4 (30°C, 4-h induction) produced in Schuffle cells. (C) Purification of DBLβ3 (30°C, 4-h induction) produced in Schuffle cells. (D) Bradford quantification of total proteins (μg/ml). Download FIG S1a, PDF file, 0.2 MB. Copyright © 2020 Joste et al. 2020 Joste et al. This content is distributed under the terms of the Creative Commons Attribution 4.0 International license.

10.1128/mBio.02103-20.3 FIG S1b Mass spectrometry analysis of recombinant PfEMP1 domains purified. Red letters are mass spectrometry (MS)-identified peptides put on expected theoretical protein sequence (black). Gray-highlighted characters represent fusion protein attached to our construction. Blue-highlighted characters represent a complete peptide obtained from MS analysis that overlaps CIDRα1.4 and DBLb3 sequences to confirm the presence of the double domain. Download FIG S1b, PDF file, 0.4 MB. Copyright © 2020 Joste et al. 2020 Joste et al. This content is distributed under the terms of the Creative Commons Attribution 4.0 International license.

10.1128/mBio.02103-20.4 FIG S2 Far-Western blot analysis. (A.1) Western blotting with an antibody against rICAM-1. (A.2) Far-Western blotting with an antibody against DBLβ3. (A.3) Far-Western blotting with an antibody against CIDRα1.4-DBLβ3. (B.1) Western blotting with an antibody against rEPCR. (B.2) Far-Western blotting with an antibody against CIDRα1.4. (B.3) Far-Western blotting with an antibody against CIDRα1.4-DBLβ3. Download FIG S2, PDF file, 0.3 MB. Copyright © 2020 Joste et al. 2020 Joste et al. This content is distributed under the terms of the Creative Commons Attribution 4.0 International license.

10.1128/mBio.02103-20.5 FIG S3 Direct ligand-receptor assay (LRA). (A) DBLβ3 on rICAM-1. (B) CIDRα1.4 on rEPCR. (C) CIDRα1.4-DBLβ3 on both rICAM-1 and rEPCR. Download FIG S3, TIF file, 0.6 MB. Copyright © 2020 Joste et al. 2020 Joste et al. This content is distributed under the terms of the Creative Commons Attribution 4.0 International license.

### Assessment of the immune response of children against PfEMP1 subdomains by ELISA.

**FIG 3:**
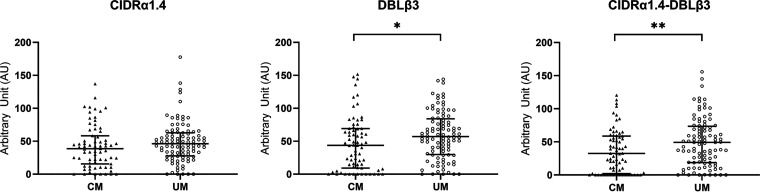
Children’s IgG levels against DBLβ3_PF3D7_1150400_, CIDRα1.4_PF3D7_1150400_, and CIDRα1.4-DBLβ3_PF3D7_1150400_. Mann-Whitney U-test was used to compare IgG levels for CM and UM patients. Values on *y* axis show ELISA arbitrary units (see Materials and Methods). Scatter plots with median and interquartile range are represented (UM, *n* = 96; CM, *n* = 71). *, *P* < 0.05; **, *P* < 0.01.

**FIG 4:**
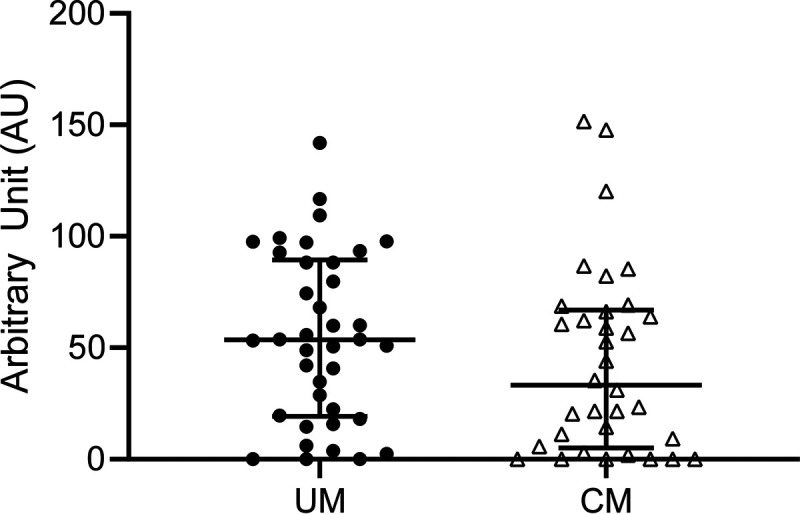
IgG level against DBLβ3 domain in CM and UM isolates expressing ICAM-1-binding DBLβ1/3 domain in RT-qPCR. Mann-Whitney U-test was used to compare IgG levels for CM and UM children. Scatter plots with median and interquartile range are represented (UM, *n* = 38; CM, *n* = 34).

A total of 71 CM and 96 UM plasma samples were tested against ICAM-1-binding motif DBLβ3_PF3D7_1150400_, CIDRα1.4_PF3D7_1150400_, and dual receptor-binding CIDRα1.4-DBLβ3_PF3D7_1150400_ recombinant domains. Significantly, higher IgG levels against DBLβ3_PF3D7_1150400_ (57 [12 to 107] arbitrary units (AU) versus 44 [0 to 103] AU) (*P* = 0.01) and CIDRα1.4-DBLβ3_PF3D7_1150400_ (49 [5 to 101] AU versus 33 [5 to 101] AU) (*P* = 0.009) domains were observed in UM children than in CM children. No difference was found for IgG against CIDRα1.4 domain (Fig. 3). Among isolates that displayed ICAM-1-binding DBLβ1/3 expression in RT-qPCR, no significant difference in IgG levels was found in CM compared to UM (Fig. 4). Moreover, no difference was observed for UM isolates that expressed or did not express ICAM-1-binding DBLβ1/3 in RT-qPCR. In the same way, UM children carrying a high CIDRα1.4 level of expression did not have a higher IgG level against CIDRα1.4_PF3D7_1150400_ than did other children (data not shown).

**FIG 5:**
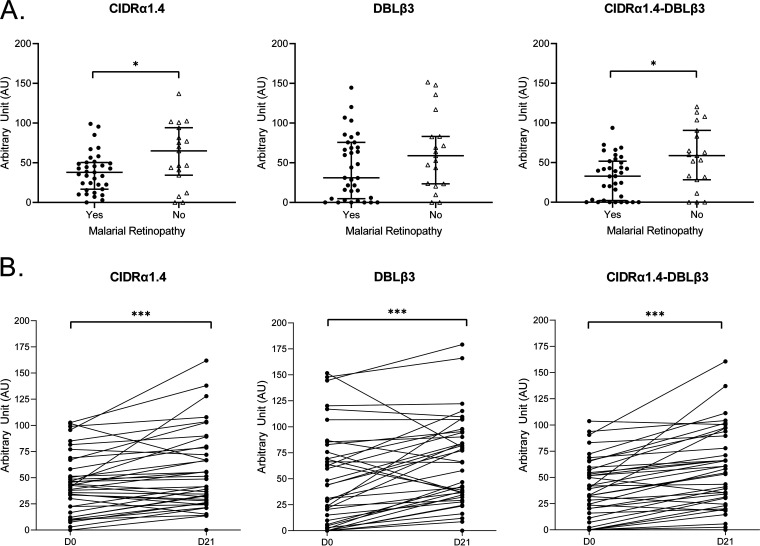
Children’s immune kinetics between D0 and D21 to D28 and comparison between malarial retinopathy and normal fundus children. (A) IgG levels of CM with normal fundus or malarial retinopathy. Mann-Whitney U-test was used to compare IgG levels between malarial retinopathy children (*n* = 35) and normal fundus children (*n* = 19). *, *P* < 0.05; **, *P* < 0.01; ***, *P* < 0.0001. (B) IgG level against CIDRα1.4_PF3D7_1150400_, DBLβ3_PF3D7_1150400_, and CIDRα1.4-DBLβ3_PF3D7_1150400_ of CM children at D0 versus D21 (*n* = 39). Wilcoxon matched-pairs signed-rank test was used to evaluate difference between D0 and D21. *, *P* < 0.05; **, *P* < 0.01; ***, *P* < 0.0001.

IgG levels were lower for children with an MR compared to normal fundus (NF) children for both CIDRα1.4_PF3D7_1150400_ (38 [10 to 65] AU versus 65 [6 to 102] AU) (*P* = 0.044) and CIDRα1.4-DBLβ3_PF3D7_1150400_ (33 [0 to 66] AU versus 59 [5 to 101] AU) (*P* = 0.028). Comparing IgG plasma levels between the day of inclusion (D0) and 21 days later (D21) for CM children, IgG levels were significantly higher in D21 plasma (Fig. 5).

## DISCUSSION

We conducted a field study in Benin and included children presenting CM and UM. We evaluated the association between *var* gene expression and *in vitro* cytoadherence to Hbec-5i and CHO-ICAM-1 cell lines as well as the prevalence of ICAM-1-binding DBLβ1/3 domain expression.

CM isolates showed higher cytoadherence levels to both cell lines than did UM isolates. Hbec-5i is a well-described but complex cellular model of CM (12, 13) expressing different membrane receptors such as EPCR, ICAM-1, and PECAM, which could explain a more pronounced variation of cytoadherence than CHO-ICAM-1. These results might be the consequence of the ability of CM isolates to bind to multiple receptors. Higher cytoadherence on CHO-ICAM-1 may result from the nature of transfected cell lines, which express more cell membrane receptors than native ones (31). Our results are consistent with a previous study from Storm et al. (18). However, Hbec-5i and CHO-ICAM-1 are not physiological models of endothelial cells. In addition, although we have validated the cytoadherence assay protocol with a selected HB3 strain, we emphasize that the experiments were performed at room temperature, which could have influenced proteins interaction at the interface between iE and the cellular model.

To investigate the higher cytoadherence capacity of CM isolates, we quantified the expression levels of *var* gene domains. As expected, we did not observe increased CD36-binding domains (CIDRα2.3/5/6/7/9/10) expression in CM isolates compared to UM isolates. Indeed, CD36-binding properties are a common feature shared by CM and UM isolates and have been rarely described as an explanatory factor in severe malaria pathophysiology (28). Then, we focused on *var* gene domains previously described as overexpressed in CM isolates analyzed by RT-qPCR (18) and transcriptome sequencing (RNA-seq) (32) techniques. CM isolates overexpressed EPCR-binding domains (CIDRα1.1, CIDRα1.4-8) and DBLα domains upstream (DBLα2/1.1/2/4/7) in accordance with the literature, confirming the major role of EPCR in CM pathophysiology (15, 17, 18). Interestingly, DBLɛ2, DBLξ3, and DBLγ1 domains were also overexpressed in CM isolates while no binding properties to endothelial receptors have been described for these domains. However, we assume that they could have an impact on the folding or maintenance of PfEMP1 three-dimensional structure as well as in IgM and α2-macroglobulin binding (33, 34). We might not focus only on domains with known endothelial receptor-binding properties to fully understand CM pathophysiology.

Surprisingly, we did not find any difference in ICAM-1-binding DBLβ1/3 domain expression in RT-qPCR between CM and UM, contrary to previous publications (18, 23). Respectively, 50% and 41% of CM and UM isolates had ICAM-1-binding DBLβ1/3 domain expression in RT-qPCR. As a confirmation, we sequenced the ICAM-1-binding motif in isolates with ICAM-1-binding DBLβ1/3 domain expression and found it in both CM and UM isolates (see Table S5 in the supplemental material). We successfully sequenced the CIDR upstream ICAM-1-binding DBLβ1/3 domain in 11 isolates (9 CM and 2 UM), all predicted to bind EPCR. These results, combined with those of RT-qPCR, pointed out that the ICAM-1-binding DBLβ1/3 domain and dual receptor-binding domains are expressed in both CM and UM isolates, questioning their role in CM physiopathology. Further studies are needed to investigate it more precisely.

In order to quantify the expression and understand the role of dual receptor-binding domains, we successfully designed primers for CIDRα1.4-DBLβ1/3, CIDRα1.6-DBLβ1/3 domains and CIDRα1.7-DBLβ1/3 domains. We had a good coverage on double domains with or without ICAM-1-binding DBLβ1/3 domains (Table S1a). We compared CM and UM isolates with no ICAM-1-binding DBLβ1/3 domain expression in RT-qPCR, and CM isolates had 5-, 13-, and 22-times higher expression of CIDRα1.4-DBLβ1/3, CIDRα1.6-DBLβ1/,3 and CIDRα1.7-DBLβ1/3, respectively, than UM isolates, suggesting a possible role of CIDRα1-DBLβ1/3 domains without ICAM-1-binding motif in CM physiopathology. Inhibition cytoadherence tests of selected P. falciparum strains with recombinant CIDRα1-DBLβ1/3 proteins with or without ICAM-1-binding motif should help to understand the role of this double domain.

To investigate the role of immune response against *var* gene domains in cerebral malaria protection, we measured IgG levels against three recombinant proteins. We hypothesized that UM children infected with parasites expressing ICAM-1-binding DBLβ1/3 domain in RT-qPCR were immunized against that domain and against dual receptor-binding domains. Surprisingly, they did not have a higher IgG level than CM children infected with parasites expressing ICAM-1-binding DBLβ1/3 domain in RT-qPCR, meaning that those UM patients did not seem to be more protected against those domains than CM patients (Fig. 4). However, we tested a single recombinant protein (PF3D7_1150400, reference sequence) for each domain, and these results must be confirmed with several recombinant proteins with various amino acid sequences.

We carried out fundus examination for each cerebral malaria patient within 1 day of inclusion (35). Of note, 67% of cerebral malaria children had MR. No difference in *var* gene expression and cytoadherence level was evidenced between children with MR and NF (Table S2), as previously described (28). Interestingly, MR children had lower IgG levels against CIDRα1.4_PF3D7_1150400_ and CIDRα1.4-DBLβ3_PF3D7_1150400_ than did NF children (Fig. 5). All together, these results suggest the role of immune response against EPCR-binding domains, such as CIDRα1.4, in MR physiopathology. Finally, we focused on the 10% of CM patients with the highest IgG level against each recombinant protein. We did not find any difference in *var* gene expression compared to the remaining CM patients.

This work showed that CM is associated with parasites presenting a higher level of cytoadherence and EPCR-binding domain expression. Contrary to previous studies, the ICAM-1-binding DBLβ1/3 domain was not overexpressed in CM isolates in comparison to UM isolates. Those isolates expressed dual receptor-binding domains as well as CM. Besides, no difference was found in IgG against DBLβ3_PF3D7_1150400_ between CM and UM isolates that express ICAM-1-binding DBLβ1/3 in RT-qPCR. To conclude, this study raises questions about the role of the A-type ICAM-1-binding domain in CM pathophysiology and finds a potential interest in CIDRα1-DBLβ1/3 double domains without the ICAM-1-binding motif.

## MATERIALS AND METHODS

### Recruitment.

**FIG 6:**
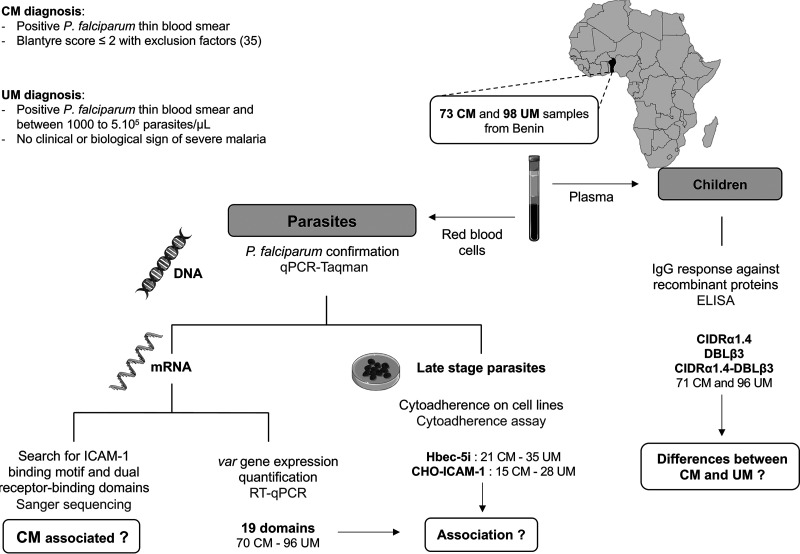
Flowchart of the study.

Ethical clearance has been obtained from Comité National d’Ethique pour la recherche en santé au Benin (N°67/MS/DC/SGM/DRFMT/CNERS/SA; 17 October 2017) and by Comité consultative de déontologie et d’éthique of Institut de Recherche pour le Développement (IRD; 24 October 2017). Patients were enrolled at Cotonou in southern Benin from December 2017 to November 2018. We included children between 2 and 6 years displaying either CM or UM. Briefly, we defined UM by fever at inclusion or within 24 h before and positive thick or thin blood smear, without clinical or biological sign of severe malaria. We defined CM by a Blantyre score at diagnosis of ≤2 and a confirmed presence of P. falciparum infection with exclusion of other causes for coma, particularly meningitis (35). Peripheral venous blood samples have been collected from all study individuals in a Vacutainer tube containing EDTA. Giemsa-stained thick blood film confirmed P. falciparum infection, and parasitemia was quantified by counting against 1,000 leukocytes. We separated plasma from total blood by centrifugation and stored it at −20°C. Ring-stage parasites were conserved in TRIzol LS reagent (Life Technologies). Finally, parasites were cultured for 18 h to 24 h *in vitro* to obtain mature forms (Fig. 6) for cytoadherence assays.

### DNA extraction.

To confirm P. falciparum infection by PCR, DNA extraction of isolates was required. Blood samples were extracted with the QIAamp DNA blood minikit (Qiagen) following the manufacturer’s instructions. Briefly, 200 μl of whole blood cells from each patient was incubated for 10 min at 56°C with 20 μl of Qiagen protease and 200 μl of lysis buffer. After incubation, 200 μl of ethanol was added and the mix was transferred on silica columns. After several washes with Qiagen buffers, DNA was eluted with 100 μl of elution buffer.

### Confirmation of Plasmodium falciparum infection and assessment of the MOI.

P. falciparum identification was confirmed by quantitative PCR (qPCR)-TaqMan (Fast-Track; Launch Diagnostic) following manufacturer’s instructions. The MOI was estimated with a fragment analysis method using the P. falciparum *msp2* polymorphic gene (36).

### RNA collection.

Plasmodium falciparum RNA preserved in TRIzol LS reagent (Life Technologies) was extracted using the phenol-chloroform method as previously described (37). Briefly, 0.2 ml chloroform was added to 1 ml blood-TRIzol sample, mixed, and then centrifuged at 4°C for 30 min at 12,000 × *g*. Supernatants were precipitated with ice-cold isopropanol and centrifuged as previously described. Pellets containing RNA were recovered with 20 μl RNase-free water. To clean our samples of any potential DNA contamination, RNA samples were digested and purified using the RNeasy minikit (Qiagen) following the manufacturer’s guidelines. qPCR on the Rotor-Gene system (Qiagen) was carried out on seryl-tRNA synthetase gene (P90 primers [38]) to assess the absence of remaining parasite genomic DNA. RNA samples were reverse transcribed to cDNA using SuperScript II (Invitrogen; Thermo Fisher Scientific Inc.).

### Cytoadherence of isolates on Hbec-5i and CHO-ICAM-1.

Static binding assays were carried out on Hbec-5i (cerebral microvascular endothelium from Homo sapiens brain, ATCC CRL-3245, USA) and Chinese hamster ovary (CHO) cell lines transfected with ICAM-1 receptor (ATCC CRL-2093, USA). Blood was centrifuged at 800 × *g* for 20 min for peripheral blood monocellular cell removal. Two hundred to four hundred microliters of ring-stage iE was matured *in vitro* during 24 to 36 h until they reached the mature stages in the first developmental cycle to avoid *var* switching (39). Briefly, isolates were cultured at 10% hematocrit in complete RPMI medium (RPMI 1640 with 0.2 mM hypoxanthine, 0.5% AlbuMAX II [Gibco], 200 μM l-glutamine [Gibco], 25 mM HEPES [Gibco], 10 μg/ml gentamicin [Gibco], and 2% human serum). Mature stages were purified using magnetic columns (MACS cell separation column; Miltenyi Biotec). All binding assays were performed in triplicate on two different petri dishes (60 × 16 mm, TPP). Thirty-five thousand cells were plated on each spot the day before the cytoadherence experiment. Fifty microliters of an iE suspension of 20% parasitemia and 1% hematocrit in binding buffer (RPMI 1640 with 3% bovine serum albumin [BSA]) was added to each spot and incubated 30 min at room temperature. Unbound erythrocytes were washed off with PBS under 30-rpm agitation on an orbital shaker. Bound erythrocytes were fixed with 1.5% glutaraldehyde in PBS for 1 h and counted microscopically. The number of iE bound to cells was determined by counting 10 fields and expressed as the number of iE bound per square millimeter. Results are expressed as the mean binding level of triplicate spots per sample.

We selected the HB3 strain on Hbec-5i and CHO-ICAM-1 cell lines as previously described (13, 40) and measured their cytoadherence value to validate this assay. We also measured the HB3-ICAM-1 cytoadherence value on CHO parental cell lines to ensure ICAM-1-binding specificity.

### *var* gene expression quantification.

Nineteen *var* gene domains were chosen based on previous publications (10, 17, 18, 32), and 122 degenerated primers were designed based on *var* gene sequences assembled (41) from whole-genome sequencing (WGS) of field isolates previously collected in 2014 and 2016 in the same geographical region (1131-Pf-BJ-Bertin, CIVIC study) (32). We evaluated our primer sensitivity on African countries from the MalariaGEN Pf3K data set (Table S1) (42). RT-qPCRs were performed using the SensiFAST Sybr No-ROX kit (Bioline) on the Rotor-Gene qPCR system (Qiagen) following these PCR conditions: 95°C for 2 s; 40 cycles of 95°C for 2 s, 50 to 60°C for 10 s, and 72°C for 10 s; and a dissociation phase from 60°C to 90°C. *var* gene transcript levels were calculated using the relative standard curve method with several plasmid dilutions (GenScript). Quantified domains were normalized with the quantification of the seryl-tRNA synthetase gene expression (38). Human and P. falciparum genomic DNAs were used as negative and positive controls, respectively, at each qPCR run.

### Sanger sequencing.

**FIG 7:**
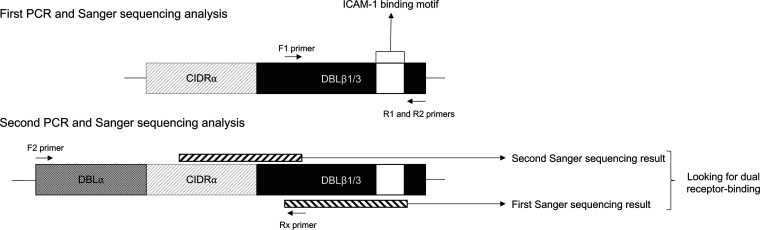
Sanger sequencing analysis of dual receptor binding. We performed PCR targeting the ICAM-1-binding motif in DBLβ1/3 domains with F1, R1, and R2 primers. We sequenced in Sanger sequencing with either R1 or R2 primer (sequence 1). On the basis of sequence 1, we designed a specific primer for each isolate. We performed the second PCR targeting CIDRα ahead of DBLβ1/3 with F2 primer (10) and the primer based on sequence 1 and sequenced with this primer. We looked for dual receptor binding on the reconstruction of CIDRα-DBLβ1/3 sequence.

We looked for the ICAM-1-binding motif (I[V/L]x3N[E]GG[P/A]xYx27GPPx3H) (23) in DBLβ1/3 RT-qPCR-positive isolates. The first PCR was performed to amplify ICAM-1-binding DBLβ1/3, and the second PCR was performed to amplify CIDRα preceding DBLβ1/3 (Fig. 7). Sanger sequencing of CIDRα-DBLβ1/3 dual receptor-binding domains was performed after purification by gel electrophoresis (NucleoSpin gel and PCR cleanup; Macherey-Nagel).

### PfEMP1 domain recombinant protein production.

Codon-optimized sequences coding for CIDRα1.4, DBLβ3, and CIDRα1.4-DBLβ3 domains from the Pf3D7_1150400 gene were synthesized for Escherichia coli (E. coli) expression (GenScript). Following the manufacturer’s guidelines, these three domains were subcloned in pET100/d-TOPO vector (Invitrogen; Thermo Fisher Scientific) and used to transform TOP10 E. coli bacteria. Purified plasmid constructions (PureLink HQ mini-plasmid purification kit; Invitrogen) were confirmed in Sanger sequencing. These plasmids were used to transform Schuffle T7 competent E. coli (New England Biolabs). Protein expression was induced by addition of 1 mM IPTG when OD_600_ reached 0.7 (in LB plus 100 μg/ml ampicillin). Then, cultures were incubated for 4 h at 30°C. Recombinant proteins were purified as previously described (43). Briefly, bacteria were incubated for 2 h at room temperature in lysis buffer (250 mM NaCl, 20 mM Tris, 1 mM DTT, 1× protease inhibitor cocktail [Roche] and 2% Sarkosyl). Samples were then sonicated and centrifuged at 3,000 × *g* for 30 min to pellet cellular fragments. Supernatants were purified on nickel-agarose resin, which specifically binds the histidine tag. Finally, proteins were eluted three times with 2 ml of buffer containing 300 mM imidazole. The purity and the specificity of all our constructions were assessed by mass spectrometry with an Orbitrap Fusion Tribrid (3P5 Cochin Facility) and SDS-PAGE/Coomassie blue staining. We tested the interaction between recombinant proteins and endothelial receptors (ICAM-1 and EPCR) in a ligand receptor assay (29) and a far-Western blotting assay (30) (Text S1).

### Mass spectrometry analysis.

Digestion was carried out using an S-trap micro spin column. Briefly, samples were heated, reduced, and alkylated at the same time for 5 min at 95°C in a buffer containing 100 mM tetraethylammonium bicarbonate, 2% SDS, 10 mM Tris(2-carboxyethyl)phosphine hydrochloride (TCEP), and 55 mM chloroacetamide. Then, samples were loaded on S-trap columns and incubated with sequencing-grade-modified trypsin (Promega) overnight at 37°C, and after digestion, peptides were eluted. Mass spectrometry analyses were performed on a Dionex U3000 RSLC nano-LC system coupled to an Orbitrap Fusion Tribrid mass spectrometer (Thermo Fisher Scientific). After drying, peptides were solubilized in 10 μl, and 1 μL was loaded, concentrated, and washed for 3 min on a C_18_ reverse-phase precolumn. Peptides were separated on a C_18_ reverse-phase resin with a 1-h gradient ending in 90% of solvent B containing 80% acetonitrile (ACN), 0.085% formic acid (FA) in H_2_O. The mass spectrometry data were analyzed using Mascot v2.5 (Matrix Science) jointly on a homemade data bank and on bacteria (333,999 sequences) from the Swiss-Prot data bank containing 560,537 sequences (July 2019). The enzyme specificity was trypsin, and up to 1 missed cleavage was tolerated. Carbamidomethylation of cysteines was set as variable modifications, and oxidation of methionines was set as fixed modifications.

### ELISA.

Enzyme-linked immunosorbent assays (ELISAs) were performed to measure the total IgG responses against the different PfEMP1 recombinant domains. Briefly, 1 μg/ml of CIDRα1.4_PF3D7_1150400_, DBLβ3_PF3D7_1150400_, or CIDRα1.4-DBLβ3_PF3D7_1150400_ recombinant proteins was applied as a coating to MaxiSorp plates (Nunc, Thermo Fisher Scientific) overnight at 4°C. After each step, wells were washed 3 times with PBS-0.1% Tween using an ELISA plate washer. Plates were blocked for 2 h at room temperature with PBS-4% BSA. Then, PBS-diluted plasma samples (1:100) were incubated for 1 h at room temperature and bound IgG was detected using a horseradish peroxidase (HRP)-conjugated mouse anti-human IgG antibody (1:5,000) [IgG(H+L) F(ab′)_2_-goat anti-human–HRP; Invitrogen]. The HRP substrate 3,3′,5,5′-tetramethylbenzidine (TMB Plus2; Eco-Tek) was added for exactly 3 min in darkness, and the reaction was stopped by adding 0.2 M H_2_SO_4_. Optical densities were measured at 450 nm using the Tecan system. ELISA arbitrary units (AU) were calculated using the following equation: AU = (OD of sample − OD of negative control)/(OD of positive control − OD of negative control) (44). The positive control was a pool of pregnant Beninese women, and the negative control was a pool of plasma from pregnant French women.

### Statistical analysis.

We compared UM and CM in terms of clinical and biological characteristics, cytoadherence, *var* gene expression, and immune response levels. Nonparametric variables were presented by median (10th to 90th percentile).

Mann-Whitney U-test was used to compare medians of clinical and biological characteristics, cytoadherence, *var* gene expression, and IgG levels and Chi-2 test was used to compare proportions of selected clinical characteristics. Correlations between cytoadherence and *var* gene expression level and between *var* gene expression levels were assessed calculating Spearman’s rank correlation coefficient rho, and *P* values were adjusted (*p-adj*) for multiple testing using the Benjamini-Hochberg correction (45). For a given cytoadherence, when several *var* gene expressions were associated (correlations with a *p-adj *of <0.35), partial correlations were calculated to control for complementary *var* gene expression (46).
