# Supplementary material for: PfEMP1 A-Type ICAM-1-Binding Domains Are Not Associated with Cerebral Malaria in Beninese Children
Source: mBio. 2020 Nov 17;11(6):e02103-20. doi: 10.1128/mBio.02103-20 (PMC7683394; doi:10.1128/mBio.02103-20)
Supplement: TABLE S1 [file mBio.02103-20-st001.pdf]

Primer Name (Domains target)	Forward Primers (5' → 3')	Reverse Primers (5' → 3')	Amplicon size bp<br>Exact Or Min - Max	Coverage CIVIC dataset<br>Number of sequences amplified with intended domain class / number of sequences with intended domain class (%)	Specificity CIVIC dataset<br>Number of sequences amplified with intended domain class / number of domain sequences amplified (%)	Specificity CIVIC dataset<br>Specificity graphic	Coverage ----- Specificity<br>Pf3K dataset
CIDRα1.1	TAGATGCCCTAAAATGGGAACATSAACT<br>TAGACAYCATAARGTGGGAACATSAACT<br>TAGACASCATAARGTGGGAATATGAACT<br>TAGACACCATAAAATGGGAACATCAACT	ACAATMHGTSACATTAGTATTATTTATGCAA<br>ACAATYGGTGYCATAATTATTATTTATGCAA<br>ACAATCCGTGACATTAGTATTATTTATACAA	65	105/117 (90%)	105/105 (100%)	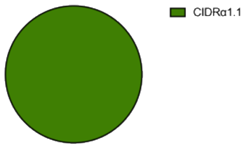	716/797 (89%)<br>-------<br>716/717 (100%)
CIDRα1.2	TAAGGGATACTATTAAGTGGAATGA<br>TAAMGGATACTATATTGTGGAATGA	CTTCATTTTTGTCTAGTTTATCCATAAC<br>CTTCTCCTTGGTTAAGATCATTCAGAAC<br>CTTCATCKTGRTTAAGCTTATCCATAACGTG	252-255	64/90 (71%)	64/65 (98%)	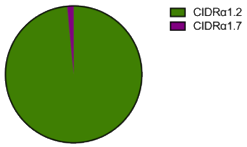	240/275 (87%)<br>-------<br>240/300 (80%)
CIDRα1.4	AATAGACAGTATAAAGTGG<br>AATAGACACTATAWADTGGG<br>AATGGATACTATAAGTTGGG	KGTTTCRTCTTGKTTAAACT<br>TGCTTCTCCTTCGTTAAACT<br>KKYTTCATCTTKGTTAAGCT	253-256	85/111 (77%)	85/92 (92%)	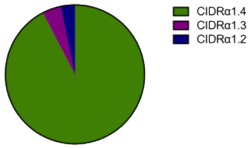	582/564 (61%)<br>-------<br>582/584 (99%)
CIDRα1.5	GAGATAMTATARWTTGGAAAAATGAMCT<br>GGGATACTATAAATTGGAAAAGCG<br>TAGACACTATAAATTGGGAAWATAAACT<br>TGGATACTACAGATTGGGATCGTA<br>ACGATACTATAGACTGGAAATACG	AAGCCAACTTTGAAAACATTTACA<br>AACCCATTKDTMAAARCATTTACA<br>AACCCATTTATCAAAACACGTACA	110-113	45/54 (83%)	45/45 (100%)	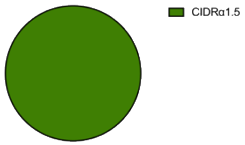	325/452 (72%)<br>-------<br>325/332 (98%)
CIDRα1.6	AAWCTATSAAAWATGGMAATGYTATTA<br>CAGCTATGAGAACTGGTTATGCTATTA	TTATTTATACAATTTGTAAGTTCGTTTTTCCA<br>ATTATTTATACAAYCCTTAAGTTCBRTTTCCCA<br>ATTATTTATACAATTCATAAGGTCATTTTT	189	32/42 (76%)	32/32 (100%)	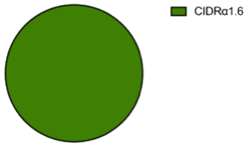	192/328 (59%)<br>-------<br>192/192 (100%)

Primer Name (Domains target)	Forward Primers (5’ → 3’)	Reverse Primers (5’ → 3’)	Amplicon size bp<br>Exact Or Min - Max	Coverage CIVIC dataset<br>Number of sequences amplified with intended domain class / number of sequences with intended domain class (%)	Specificity CIVIC dataset<br>Number of sequences amplified with intended domain class / number of domain sequences amplified (%)	Specificity CIVIC dataset<br>Specificity graphic	Coverage ----- Specificity<br>Pf3K dataset
CIDRα1.7	CGGAAACTATAAGGTGGAACGATAA<br>CGGAAACTATAACGTGGAAMGATAA<br>AGGATACTATAWTGTGGAATGATAA	AATAGTTCCTTTATACTATTCCATTC<br>AATAGTTTCTTTATACTACTCCATTC<br>AATAGTTTCTTTATACTATTCCATTC<br>AATARTTYCTTTATATTATTCCATTC	138 - 141	63/74<br>(84%)	63/73<br>(86%)	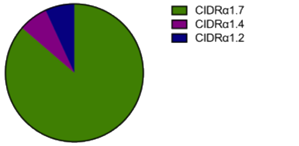	474/636<br>(75%)<br>-------<br>474/474<br>(100%)
CIDRα1.8	TATTAATAGACAYCATAAARTGGGAA<br>TATTAATAGACAGTATAATGTGGGAA<br>TAATAAAGGATACTATAAAGTGGGAA	ACAATTSTTGTTACATTYATTATMACAAT<br>ACAATTTTCGTTACAGCCATTRTYACAAT	95	44/50<br>(88%)	44/44<br>(100%)	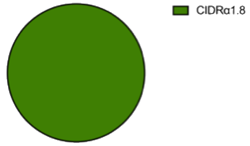	248/305<br>(81%)<br>-------<br>248/253<br>(98%)
CIDRα2.3/5/6/<br>7/9/10	TTYTTTTGGRATKGGGTACATGATATGTTAM<br>TTTTTTTGGRAKTGGGTWTATSRTATGTTAC<br>TTTTTTTGGAAGTGGGTTACCGAAATGTTAA	AAGTTSTSTCTTCCACTCTACAGAATC<br>AAGTTSBTTTCTCCAMTCTAMAGAATC<br>AAGTTCTTTCCTCCAATMYATAGAATC<br>AWGTTCGTTTCTCCATTTAATAGAATS	60	251/460<br>(55%)	251/260<br>(97%)	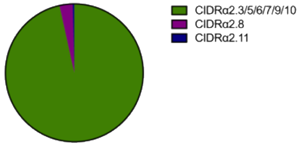	1603/3084<br>(52%)<br>-------<br>1603/1630<br>(98%)
CIDRδ-Nter	TAAATGTAACTTACATGTATGTGAAC<br>TAAATGTWACTTAGAYGTATGTGAAC<br>TAAATGTAAGTTAGATGTGTGTGAAC<br>TAAATGTAAYATAAATGTATGTGAAY<br>TAAATGTAATTTASATGTATGTGAAC	AATACTTTAACCAACGYTTARTCAATAC<br>AATATTCTAACCACCGTTTAAATAGTAC<br>AATACATCAACCAACGCTTAATCAATAC<br>AATACTGYAACCAACGTTTAATCAATAC<br>AAKRCTCTAWCCAACGTTTAATRAATAC	104	124/157<br>(78%)	124/124<br>(100%)	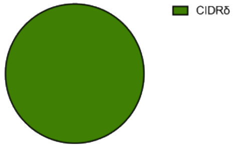	398/555<br>(72%)<br>-------<br>398/403<br>(99%)
DBLα2/1.1/1.2/<br>1.4/1.7	GAYTATGTYCCTCAATWTTTACGTTGGT<br>GATTACGTNCCTCAATWTTTACGBTGGT<br>GATTACGTHCCTCAATWTTTAAGATGGT<br>GACTAYGTYCCTCAAYWTTTAMGWTGGT	NTACAATCATATCCATYATGWCWACAA<br>GTACAATCAWATCCATTCTGACTACAA<br>KTACARTCAWATCCATTAARACTACAA	122	325/599<br>(54%)	325/343<br>(95%)	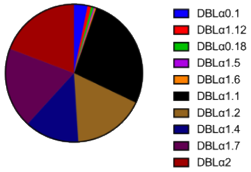	1976/3641<br>(54%)<br>-------<br>1976/2009<br>(98%)

Primer Name (Domains target)	Forward Primers (5' → 3')	Reverse Primers (5' → 3')	Amplicon size bp<br>Exact Or Min - Max	Coverage CIVIC dataset<br>Number of sequences amplified with intended domain class / number of sequences with intended domain class (%)	Specificity CIVIC dataset<br>Number of sequences amplified with intended domain class / number of domain sequences amplified (%)	Specificity CIVIC dataset<br>Specificity graphic	Coverage ----- Specificity<br>Pf3K dataset
DBLα1.7	AATATAGACATCCTTGTGCTCG<br>AGGATAGACATCCTTGTCGTTT<br>MATATAGRCATCCTTGYGCTGG<br>GTGAAAGAGATCCTTGTTATCG	ACATACAGCTCCTTCCAAATTAAACA<br>ACATATTGCATCTCCTTTATCTGAAAA<br>ACATTCTGCTTCACYTTCRTWTGAAAA<br>TACATACTGCTCCTTCCAAATTCGGATT	64 - 65	66/116<br>(58%)	67/70<br>(96%)	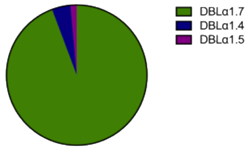	311/746<br>(42%)<br>-------<br>311/327<br>(95%)
DBLβ1/3	TACAAAVAMGCAGAARTTTATGCTS<br>TACGCAAAAGCACGAATTRYTGCTA	TTATAATACCCAGWACCRCCATT	50	71/81<br>(87% of DBLβ1 or DBLβ3 with ICAM-1 binding motif)	71/77<br>(92%)	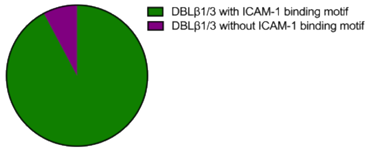	489/595<br>(82%)<br>-------<br>489/595<br>(82%)
DBLβ5	TGGATGAMNGAATGGGHCGAATGGT<br>TAAGATGGATGAATGAATGGGCWGAATG<br>TGGATGTCCGAATGGGCAGAATGGT<br>TGGTTAACMGAATGGKCCGAATGGTTC	TAATTBTTGTAAGAAAGCTACCAC<br>TATTGGGGTCAAAAAATCTACCAC<br>GGAGTAATTCTTGTAAAAATTTAACCAC	325 - 346	58/223<br>(26%)	58/68<br>(85%)	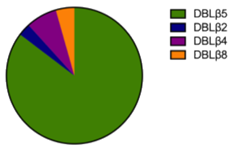	314/3403<br>(9%)<br>-------<br>314/357<br>(88%)
DBLε2	WAATTTAATAGGTTTGGAAGCAYAY<br>AAMWTTAATWGGTTTGGGAGCAC<br>ARATTTRATWGGTTTAAATGCAYAC<br>AAAATTAATAGGTTTAGAAGCACAC	GRCATAATTGTYGTACTCTAGGWGRMA<br>GRCATAAYTGMTBATCTCTAGGAGAAA<br>AAYATAATTGTTGTACTCTAGGAGAAA<br>GACATAATTGTTGCACTCTAGGAGRMA	86 - 92	40/64<br>(63%)	40/40<br>(100%)	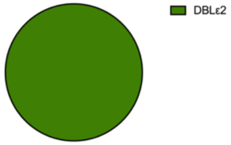	325/595<br>(55%)<br>-------<br>325/327<br>(99%)
DBLγ1	AGCTTTYATCAAATCTGCAGCAGCAGAAACA<br>AGCATTCATCSAATGTKCWGCAGCAGAAACT	AAAAAATCTCTATAATCTCCAAAYGTGTAGAACAT<br>CATAAATCTCTATAATCACCAAATGTGTAGAACAT	153 - 228	51/112<br>(46%)	51/51<br>(100%)	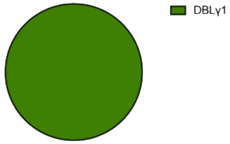	62/335<br>(78%)<br>-------<br>62/65<br>(95%)

Primer Name (Domains target)	Forward Primers (5' → 3')	Reverse Primers (5' → 3')	Amplicon size bp<br>Exact Or Min - Max	**Coverage** CIVIC dataset<br>Number of sequences amplified with intended domain class / number of sequences with intended domain class (%)	**Specificity** CIVIC dataset<br>Number of sequences amplified with intended domain class / number of domain sequences amplified (%)	**Specificity** CIVIC dataset<br>Specificity graphic	**Coverage** ----- **Specificity**<br>Pf3K dataset
DBLξ3	AYCCCCCYGTTGWTGAYTATATYCCWCA<br>ATCCACCTGATAATGATTACATTCCACA<br>DTCCTCCTTAYGATGATTATATWCCWCA<br>AACCTCCTTATGWYGAYTAYATTCCWCA	TTCTGACCATTCAGTCATCCATCT<br>YTCTCCCCATTCCTTCATCCATCT<br>TTCMCTCCATTCAGTYAWCCAACG<br>TTCACTCCATTCTTGCATCCAWCG	59	65/97<br>(67%)	65/65<br>(100%)	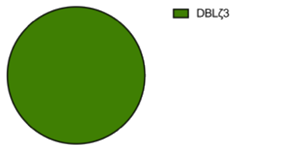	387/621<br>(62%)<br>-------<br>387/391<br>(99%)
CIDRα1.4-DBLβ1/3	TGGAATAAACTTAAGGAAAATTTAAAARAAAAAAT<br>AACTTAAAGAAAATTTAAAAAAGCAAATTGCG<br>TGGAWGAACTTAAACAAAAWTTARAARAAAAAAT	CGTTTATAATATTGTGCKATTTGTTTYAC<br>CGTTTAAGGTATTGTGCTATTTGTTTYAC<br>CKTTTAAARTATTGTGCTATTTGTTTYAC	238 - 266	60/99<br>(61%)<br><br>28/40 (70%)<br>DBLβ1/3 with ICAM-1-binding motif<br><br>32/59 (54%)<br>DBLβ1/3 without ICAM-1-binding motif	60/68<br>(88%)	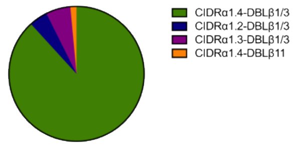	337/ 826<br>(41%)<br>-------<br>337/368<br>(92%)
CIDRα1.6-DBLβ1/3	AAAAGATATAGCTGAAAGATGCATAGACAATAA<br>AAAAGAAACTGCAACAATATGCAAAGATAATAA	CGTTTATAATATTGTGCTATTTGTTTYAC<br>TKTTGCATATSTTCHGCTARTTGTTTTAC	144 - 156	21/26<br>(81%)<br><br>6/8 (75%)<br>DBLβ1/3 with ICAM-1-binding motif<br><br>15/18 (83%)<br>DBLβ1/3 without ICAM-1-binding motif	21/21<br>(100%)	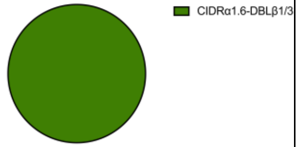	146/258<br>(57%)<br>-------<br>146/163<br>(90%)
CIDRα1.7-DBLβ1/3	GAATGGAATAGTATAAAGGAACTATT<br>GAATGGAGTAGTATAAAGAAACTATT<br>GAATGGAATAGTATAAAGAAACTATT<br>GAATGGAATAATATAAAGRAAYTATT	CKTTTATAATATTGTGCKAYTTGTTTTAC<br>CKTTTAAAGTATTGTGCTATTTGTTTYAC<br>CGTTTATAATATTGTGCKATTTGTTTCAC	377 - 401	50/64<br>(78%)<br><br>10/15 (67%)<br>DBLβ1/3 with ICAM-1-binding motif<br><br>40/49 (82%)<br>DBLβ1/3 without ICAM-1-binding motif	50/69<br>(72%)	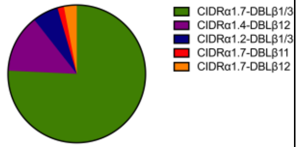	316/495<br>(64%)<br>-------<br>316/382<br>(83%)

Supplemental table S1a - *In silico* newly designed RT-qPCR *var* gene primers based on CIVIC dataset
Coverage and specificity values were calculated on CIVIC and Pf3K (African countries) datasets

Name	Primer
F1-primer	5’-AAACAAATMGCACAATAYYWTAAA-3’
F2-primer	5’-GCAMGMAGTTTYGCNGATATWGG-3’
R1-primer	5’-TGTAYATATCCTTCAGCAGTACTATA-3’
R2-primer	5’-TGTATATATCCTTCAGGAGTACTATA-3’

Supplemental table S1b - *In silico* designed primers used in Sanger sequencing
