# Supplementary material for: PfEMP1 A-Type ICAM-1-Binding Domains Are Not Associated with Cerebral Malaria in Beninese Children
Source: mBio. 2020 Nov 17;11(6):e02103-20. doi: 10.1128/mBio.02103-20 (PMC7683394; doi:10.1128/mBio.02103-20)
Supplement: TABLE S2 [file mBio.02103-20-st002.docx]

| Domain | Group | Predicted receptor | Malarial retinopathy | | Normal fundus | |
| --- | --- | --- | --- | --- | --- | --- |
|  |  |  | n | Median | n | Median |
| CIDRα1.1 | B/A | EPCR | 34 | 0.076 (0-1.17) | 16 | 0.060 (0-0.44) |
| CIDRα1.8 | B/A | EPCR | 35 | 0.00053 (0-0.027) | 16 | 0.00065 (0-0.13) |
| CIDRα1.4 | A | EPCR | 35 | 0.26 (0-4.9) | 16 | 0.056 (0-2.2) |
| CIDRα1.5 | A | EPCR | 35 | 0.0058 (0.00016-0.045) | 16 | 0.0085 (6.10^-5^-0.072) |
| CIDRα1.6 | A | EPCR | 35 | 0.0096 (0-0.14) | 16 | 0.0055 (0.00011-0.056) |
| CIDRα1.7 | A | EPCR | 35 | 0.0070 (0.00019-0.051) | 16 | 0.013 (0.00022-0.12) |
| CIDRα1.2 |  |  | 34 | 0.0076 (0-0.025) | 16 | 0.0070 (4.10^-5^-0.049) |
| CIDRδ | A |  | 34 | 0.0074 (0-0.40) | 16 | 0.0078 (0-0.13) |
| CIDRα2.3/5/6/7/9/10 | B | CD36 | 34 | 0.00082 (0-0.0066) | 16 | 0.00030 (4.10^-6^-0.0018) |
| DBLα1.7 | A |  | 33 | 0.19 (0.0054-11) | 16 | 0.77 (0-6.9) |
| DBLα2/1.1/2/4/7 | A |  | 35 | 0.35 (0.096-2.50) | 16 | 0.44 (0.052-1.40) |
| DBLβ1/3-motif | A | ICAM-1 | 34 | 0.0014 (0-0.30) | 16 | 0 (0-0.23) |
| DBLβ5 | B | ICAM-1 | 35 | 0.039 (0.00010-0.21) | 16 | 0.0074 (0.00031-0.077) |
| DBLε2 |  |  | 35 | 0.00014 (0-0.0050) | 16 | 0 (0-0.0025) |
| DBLξ3 |  |  | 35 | 0.0028 (0-0.019) | 16 | 0.0019 (0-0.014) |
| DBLγ1 |  |  | 35 | 0.0017 (0.00029-0.0049) | 16 | 0.00070 (4.10-6-0.032) |
| CIDRα1.4-DBLβ1/3 | A | EPCR +/- ICAM-1 | 35 | 0.041 (0-0.35) | 16 | 0.012 (0.0025-0.11) |
| CIDRα1.6-DBLβ1/3 | A | EPCR +/- ICAM-1 | 35 | 0.018 (0-0.083) | 16 | 0.0052 (0-0.18) |
| CIDRα1.7-DBLβ1/3 | A | EPCR +/- ICAM-1 | 33 | 0.058 (0.00090-0.58) | 15 | 0.080 (0.0028-0.64) |

**Supplemental table S2** - Transcripts levels of var genes and cytoadherence value between malaric retinopathy and normal fundus isolates. Results are expressed after normalization with P90 quantification. Results shown are median with 10^th^ and 90^th^ percentile. P-value were calculed with Mann-Whitney U-test. Number of isolates (n) in the analysis is specified. None of the median comparison was statistically significant.
