# Supplementary material for: PfEMP1 A-Type ICAM-1-Binding Domains Are Not Associated with Cerebral Malaria in Beninese Children
Source: mBio. 2020 Nov 17;11(6):e02103-20. doi: 10.1128/mBio.02103-20 (PMC7683394; doi:10.1128/mBio.02103-20)
Supplement: TABLE S4 [file mBio.02103-20-st004.docx]

|  | n | *var* domain | Adjustement variable | Correlation | p-value |
| --- | --- | --- | --- | --- | --- |
| CM population | | | | | |
| CHO | 14  14  14 | CIDRα1.4  DBLβ1/3  DBLε2 | DBLβ1/3, DBLε2  CIDRα1.4, DBLε2  CIDRα1.4, DBLβ1/3 | 0,50  0,52  -0,45 | 0.10  0.08  0.14 |
| UM population | | | | | |
| CHO | 26  26  26  26 | CIDRα1.4-DBLβ1/3  CIDRα1.6  CIDRα1.2  DBLα1.7 | CIDRα1.6, CIDRα1.2, DBLα1.7  CIDRα1.4-DBLβ1/3, CIDRα1.2, DBLα1.7  CIDRα1.4-DBLβ1/3, CIDRα1.6, DBLα1.7  CIDRα1.4-DBLβ1/3, CIDRα1.6, CIDRα1.2 | 0.49  -0.53  -0.31  0.15 | 0.02  0.009  0.15  0.50 |

**Supplemental table S4** **–** **Spearman partial correlation**. As none univariate correlation was significative for CM population and only one for UM population, partial correlation was not calculated for Hbec-5i cytoadherence.
