# Supplementary material for: PfEMP1 A-Type ICAM-1-Binding Domains Are Not Associated with Cerebral Malaria in Beninese Children
Source: mBio. 2020 Nov 17;11(6):e02103-20. doi: 10.1128/mBio.02103-20 (PMC7683394; doi:10.1128/mBio.02103-20)
Supplement: TEXT S1 [file mBio.02103-20-s0001.docx]

**Supplemental Methods**

**1. Digestion of recombinant PfEMP1 domains produced.** Tryptic digestion and mass spectrometry analysis were performed by the proteomic platform 3P5 (Université de Paris, Institut Cochin, INSERM, U1016, CNRS, UMR8104, F-75014 PARIS, France). Digestion was carried out using S-Trap Micro Spin Column Digestion protocol from Protifi company. Briefly, samples were heated, reduced and alkylated at the same time for 5 minutes at 95°C in a buffer containing TetraEthylAmmonium Bicarbonate 100 mM, SDS 2%, 10 mM TCEP and 55 mM chlorodoacetamide. Then, samples were loaded on S-trap columns and incubated with sequencing grade-modified trypsin (Promega, USA) overnight at 37 °C and after digestion peptides were eluted from columns. Extracts were dried using a vacuum centrifuge concentrator plus (Eppendorf).

**2. Mass spectrometry analysis of recombinant PfEMP1 domains produced.** Mass spectrometry (MS) analyses were performed on a Dionex U3000 RSLC nano-LC system coupled to an Orbitrap Fusion Tribrid mass spectrometer (Thermo Fisher Scientific). After drying, peptides were solubilized in 10 µL of 0.1 % trifluoroacetic acid (TFA) containing 10 % acetonitrile (ACN). One µL was loaded, concentrated and washed for 3 min on a C18 reverse phase precolumn (3 µm particle size, 100 Å pore size, 75 µm inner diameter, 2 cm length, Thermo Fisher Scientific). Peptides were separated on a C18 reverse phase resin (2 µm particle size, 100 Å pore size, 75 µm inner diameter, 25 cm length from Thermo Fisher Scientific) with a 1 hour gradient starting from 99 % of solvent A containing 0.1 % FA in H2O and ending in 90 % of solvent B containing 80 % ACN, 0.085 % FA in H2O. The mass spectrometer acquired data throughout the elution process and operated in a data-dependent scheme with full MS scans acquired with the Orbitrap, followed by MS/MS HCD fragmentations acquired with the Ion Trap on the most abundant ions detected in top speed mode for 3 seconds. Resolution was set to 60,000 for full scans at AGC target 2.0e5 within 60 ms maximum injection ion time (MIIT). The MS scans spanned from 350 to 1500 m/z. Precursor selection window was set at 1.6 m/z, and MS/MS scan resolution was set with AGC target 2.0e4 within 100 ms MIIT. HCD Collision Energy was set at 30 %. Dynamic exclusion was set to 30 s duration. For the spectral processing, the software used to generate .mgf files was Proteome Discoverer 1.4 (ThermoFisher Scientific). The mass spectrometry data were analyzed using Mascot v2.5 (Matrix science) jointly on a homemade databank and on bacteria (333,999 sequences) from the SwissProt databank containing 560,537 sequences; 201,466,755 residues (July 2019). The enzyme specificity was Trypsin’s and up to 1 missed cleavage was tolerated. The precursor mass tolerance was set to 4 ppm and the fragment mass tolerance to 0.55 Da for Fusion data. Carbamidomethylation of cysteins was set as variable modifications and oxidation of methionines was set as fixed modifications.

**3. Assessment of recombinant domains binding activity on EPCR and ICAM1 receptor**

Recombinant proteins binding capacity (DBLβ3, CIDRα1.4 and CIDRα1.4-DBLβ3) were assessed with Far western Blotting and ELISA.

Far western blots were performed following the protocol described by Wu *et al* (1). Briefly, 2 µg of ICAM-1 and EPCR recombinant proteins (R&D System™ Bio-Techne SAS) were loaded in duplicates onto a 10% acrylamide gel and separated by SDS-PAGE. Proteins were then transferred onto a nitrocellulose membrane that was separated in two identical membranes. The first membrane was used for western blot analysis to detect ICAM-1 and EPCR (anti-ICAM1 clone OTI2H4 Origene™ and anti-EPCR polyclonal Thermo Fisher™). The second membrane was used for the Far western Blot to detect ligand proteins (DBLβ3, CIDRα1.4 and CIDRα1.4-DBLβ3). As it is essential for the binding activity that proteins refold correctly, the second membrane was incubated with gradually reduced guanidine buffer (6M – 30min ; 3M – 30min; 1M – 30min; 0.1M – 30min at 4°C and guanidine-free buffer overnight at 4°C). Far western membranes were blocked with 5% skim milk for 1h at room temperature (RT) and then incubated with 35 µg of associated recombinant proteins at 4°C overnight. After that, membranes were incubated with a primary antibody (anti-tag Xpress antibody, Thermo Fisher Scientific™) against recombinant proteins overnight at 4°C. After several washes with PBST buffer, membranes were incubated with a secondary antibody targeting mouse IgG coupled with a 680nm dye (F(ab')2-Goat anti-Mouse IgG (H+L) Secondary Antibody, Alexa Fluor 680, Invitrogen™) and finally revealed with Odyssey® Fc Imaging System (LI-COR Biosciences). Classic and far western blot were compared to assess the binding capacity of the bait proteins on ICAM-1 receptor and/or EPCR.

We also assessed the binding capacity of the recombinant proteins on their specific receptors by ELISA using a direct Ligand-Receptor Assay (LRA) (2). Plates were coated with 100 µL of 2 µg/mL EPCR and/or 0.1 µg/mL of ICAM-1 recombinant proteins (R&D System™ Bio-Techne SAS) at 4°C overnight. Plates were blocked with 5% BSA for 2h at RT and then incubated 2h at RT with different concentrations of DBLβ3, CIDRα1.4 or CIDRα1.4-DBLβ3 (0; 0.031 ; 0.063; 0.125; 0.250; 0.500; 1; 2; 4 and 8µg/mL). Primary antibodies (Xpress antibody, Thermo Fisher Scientific™) were incubated for 2h at RT and HRP coupled secondary antibody (IgG (H+L) Poly-HRP Goat anti-Mouse, HRP, Invitrogen™) was incubated for 1h at RT. Then 100 µL of TMB ([TMB PLUS2™, ECO-TEK®](https://www.clinisciences.com/autres-produits-186/tmb-plus2-eco-tek-538000012.html)) was added and the reactions were stopped after 3 minutes with 100 µL of 0.2M H_2_SO_4_. Optical density (OD) was measured at 450 nm with the Infinite® F50 TECAN system. Background signal was measured for each ligand concentration and subtracted from the measured OD and results were expressed as binding percentage.

**References**

1. Wu Y, Li Q, Chen X-Z. 2007. Detecting protein-protein interactions by Far western blotting. Nat Protoc 2:3278–3284.

2. Syedbasha M, Linnik J, Santer D, O’Shea D, Barakat K, Joyce M, Khanna N, Tyrrell DL, Houghton M, Egli A. 2016. An ELISA Based Binding and Competition Method to Rapidly Determine Ligand-receptor Interactions. J Vis Exp.
