# Supplementary material for: PfEMP1 A-Type ICAM-1-Binding Domains Are Not Associated with Cerebral Malaria in Beninese Children
Source: mBio. 2020 Nov 17;11(6):e02103-20. doi: 10.1128/mBio.02103-20 (PMC7683394; doi:10.1128/mBio.02103-20)
Supplement: FIG S1a [file mBio.02103-20-sf01a.pdf]

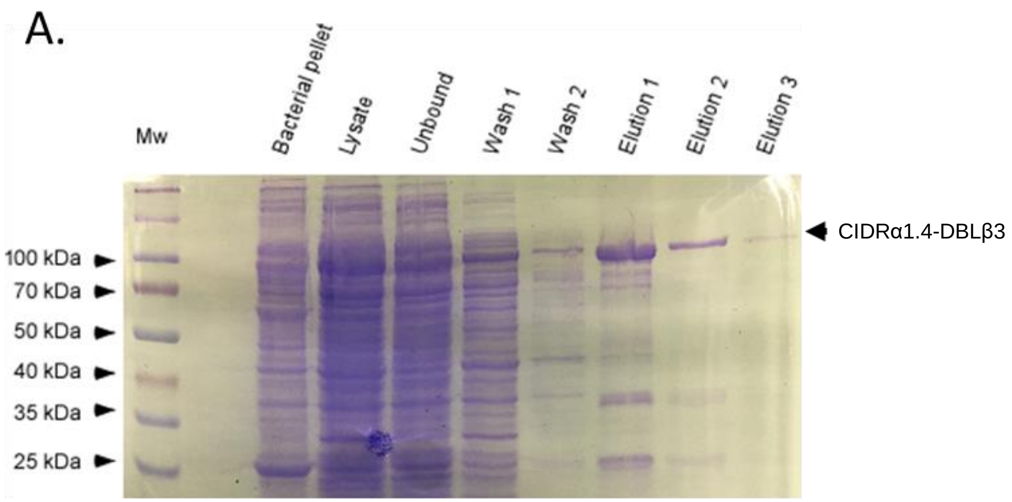

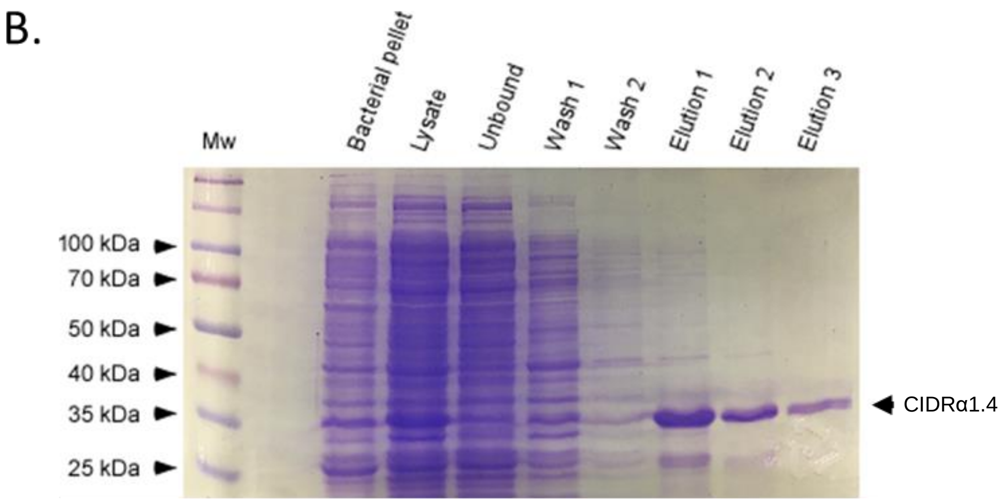

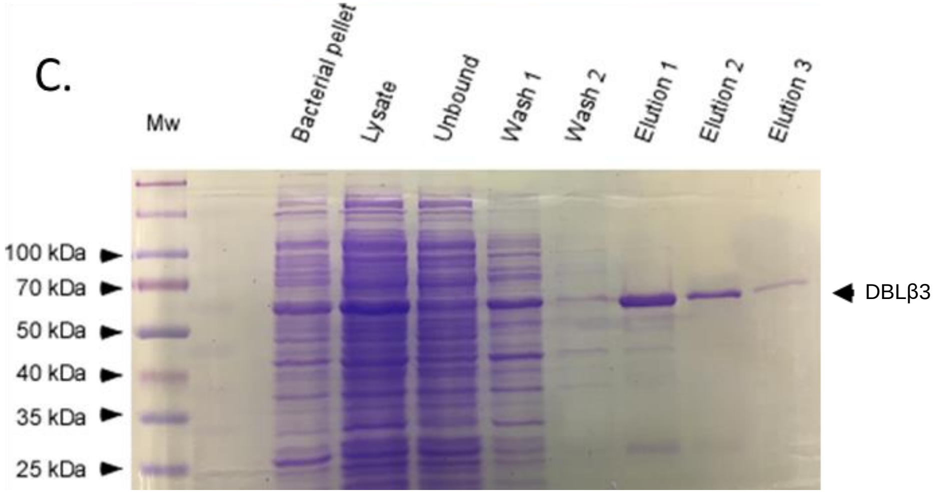

D.

	DBLβ3	CIDRα1.4	CIDRα1.4-DBLβ3
Elution 1	177	626	386
Elution 2	46	276	76
Elution 3	23	85	18

Supplemental figure S1 a : SDS-PAGE & Coomassie blue staining of different recombinant PfEMP1 domains purification. **A:** Purification of CIDRα1.4-DBLβ3 (30°C-4h induction) produced in Schuffle cells. **B:** Purification of CIDRα1.4 (30°C-4h induction) produced in Schuffle cells. **C:** Purification of DBLβ3 (30°C-4h induction) produced in Schuffle cells. **D:** Bradford quantification of total protein (µg/mL).
