# Supplementary material for: PfEMP1 A-Type ICAM-1-Binding Domains Are Not Associated with Cerebral Malaria in Beninese Children
Source: mBio. 2020 Nov 17;11(6):e02103-20. doi: 10.1128/mBio.02103-20 (PMC7683394; doi:10.1128/mBio.02103-20)
Supplement: FIG S1b [file mBio.02103-20-sf01b.pdf]

>MS results of purified CIDRα1.4 domain (68% coverage; Score: 7365)
MRGSHHHHHH GMASMTGGQQ MGRDLYDDDD KDHPFWDCGV DCSSGTCIEK
KDDINCGKKI NYEPPHGVKP IDIIVLYSGN EEGEITKRLS EFCTDSSNNK
GKNYEQWKCY YKNGDDNKCK MVKNSGNNIT EEKIISFDEF FYVWVRKLLI
DSIKWENELN NCIDNTSTHC NKECNKNCEC FDKWVKKKED EWKNVKNVFE
NKNGTSHNYY NKLNGLFKGF FFEVMDKLNK DETKWNKLIE SLRTKIDSSK
ENIGTGNTQD TIKVLLDHLK ETATICKDNN TNEA

>MS results of purified CIDR-DBL 18C domain (65% coverage; Score: 13179)
MRGSHHHHHH GMASMTGGQQ MGRDLYDDDD KDHPFWDCGV DCSSGTCIEK
KDDINCGKKI NYEPPHGVKP IDIIVLYSGN EEGEITKRLS EFCTDSSNNK
GKNYEQWKCY YKNGDDNKCK MVKNSGNNIT EEKIISFDEF FYVWVRKLLI
DSIKWENELN NCIDNTSTHC NKECNKNCEC FDKWVKKKED EWKNVKNVFE
NKNGTSHNYY NKLNGLFKGF FFEVMDKLNK DETKWNKLIE SLRTKIDSSK
ENIGTGNTQD TIKVLLDHLK ETATICKDNN TNEACETSRN RKTNPCAKPH
GKKLATVKQI AQYYKRKAYI QLNERGSRSA LKGDASQGQY DRGGKADDFK
TKLCEINEKH SNARSNSLNP CNGKDNNKVR FNVGTPWQSG EKIATATDVY
LPPRRQHFCT SNLEYLINGG HQAILNVKNG KINHSFLGDV LLAAKYQAQH
TMKDYKSKND KEGICRAIRY SFADIGDIIK GTDLWDKDGG EIKTQNHLVT
IFDKIKAQLP KDIKGKYTGT KHLELRKDWW EANRDQVWKA MQCGNDNPCS
GESDHTPLHD YIPQRLRWMT EWAEWYCKEQ SRLYDKLKVC EECMRKGESC
TKGSGECATC KEACEEYNKE IKKWEQQWDA ISYKYLMLYA KARITAINGG
PGYYNTEVQE EDKPVVDFLY NLYLQNGGKK GPPPDTHRVK ALIARVKRDA
ARNRVKRADG SSATRVTATT TITPYSTAAG YIHQEAHIGD CQKQTQFCKN
KNGSDVSDTE ADPTYAFRDK PHDHDTACKC

>MS results of purified CIDR-DBL 30C domain (72% coverage; Score: 11094)
MRGSHHHHHH GMASMTGGQQ MGRDLYDDDD KDHPFWDCGV DCSSGTCIEK
KDDINCGKKI NYEPPHGVKP IDIIVLYSGN EEGEITKRLS EFCTDSSNNK
GKNYEQWKCY YKNGDDNKCK MVKNSGNNIT EEKIISFDEF FYVWVRKLLI
DSIKWENELN NCIDNTSTHC NKECNKNCEC FDKWVKKKED EWKNVKNVFE
NKNGTSHNYY NKLNGLFKGF FFEVMDKLNK DETKWNKLIE SLRTKIDSSK
ENIGTGNTQD TIKVLLDHLK ETATICKDNN TNEACETSRN RKTNPCAKPH
GKKLATVKQI AQYYKRKAYI QLNERGSRSA LKGDASQGQY DRGGKADDFK
TKLCEINEKH SNARSNSLNP CNGKDNNKVR FNVGTPWQSG EKIATATDVY
LPPRRQHFCT SNLEYLINGG HQAILNVKNG KINHSFLGDV LLAAKYQAQH
TMKDYKSKND KEGICRAIRY SFADIGDIIK GTDLWDKDGG EIKTQNHLVT
IFDKIKAQLP KDIKGKYTGT KHLELRKDWW EANRDQVWKA MQCGNDNPCS
GESDHTPLHD YIPQRLRWMT EWAEWYCKEQ SRLYDKLKVC EECMRKGESC
TKGSGECATC KEACEEYNKE IKKWEQQWDA ISYKYLMLYA KARITAINGG
PGYYNTEVQE EDKPVVDFLY NLYLQNGGKK GPPPDTHRVK ALIARVKRDA
ARNRVKRADG SSATRVTATT TITPYSTAAG YIHQEAHIGD CQKQTQFCKN
KNGSDVSDTE ADPTYAFRDK PHDHDTACKC

>MS results of purified DBLβ3 domain (69% coverage; Score: 8060)
MRGSHHHHHH GMASMTGGQQ MGRDLYDDDD KDHPFWRNRK TNPCAKPHGK
KLATVKQIAQ YYKRKAYIQL NERGSRSALK GDASQGQYDR GGKADDFKTK
LCEINEKHSN ARSNSLNPCN GKDNNKVRFN VGTPWQSGEK IATATDVYLP
PRRQHFCTSN LEYLINGGHQ AILNVKNGKI NHSFLGDVLL AAKYQAQHTM
KDYKSKNDKE GICRAIRYSF ADIGDIIKGT DLWDKDGGEI KTQNHLVTIF
DKIKAQLPKD IKGKYTGTKH LELRKDWWEA NRDQVWKAMQ CGNDNPCSGE
SDHTPLHDYI PQRLRWMTEW AEWYCKEQSR LYDKLKVCEE CMRKGESCTK
GSGECATCKE ACEEYNKEIK KWEQQWDAIS YKYLMLYAKA RITAINGGPG
YYNTEVQEED KPVVDFLYNL YLQNGGKKGP PPDTHRVKAL IARVKRDAAR
NRVKRADGSS ATRVTATTTI TPYSTAAGYI HQEAHIGDCQ KQTQFCKNKN
GSDVSDTEAD PTYAFRDKPH DHDTACKC

Supplemental Figure S1a. Mass spectrometry analysis of recombinant PfEMP1 domains purified. Red letters are MS-identified peptides put on expected theoretical protein sequence (black). Grey highlighted characters represent fusion protein attached to our construction. Blue highlighted characters represent a complete peptide obtained from MS analysis that overlap CIDRα1.4 and DBLβ3 sequences to confirm the presence of the double domain.
