# Supplementary material for: PfEMP1 A-Type ICAM-1-Binding Domains Are Not Associated with Cerebral Malaria in Beninese Children
Source: mBio. 2020 Nov 17;11(6):e02103-20. doi: 10.1128/mBio.02103-20 (PMC7683394; doi:10.1128/mBio.02103-20)
Supplement: FIG S2 [file mBio.02103-20-sf002.pdf]

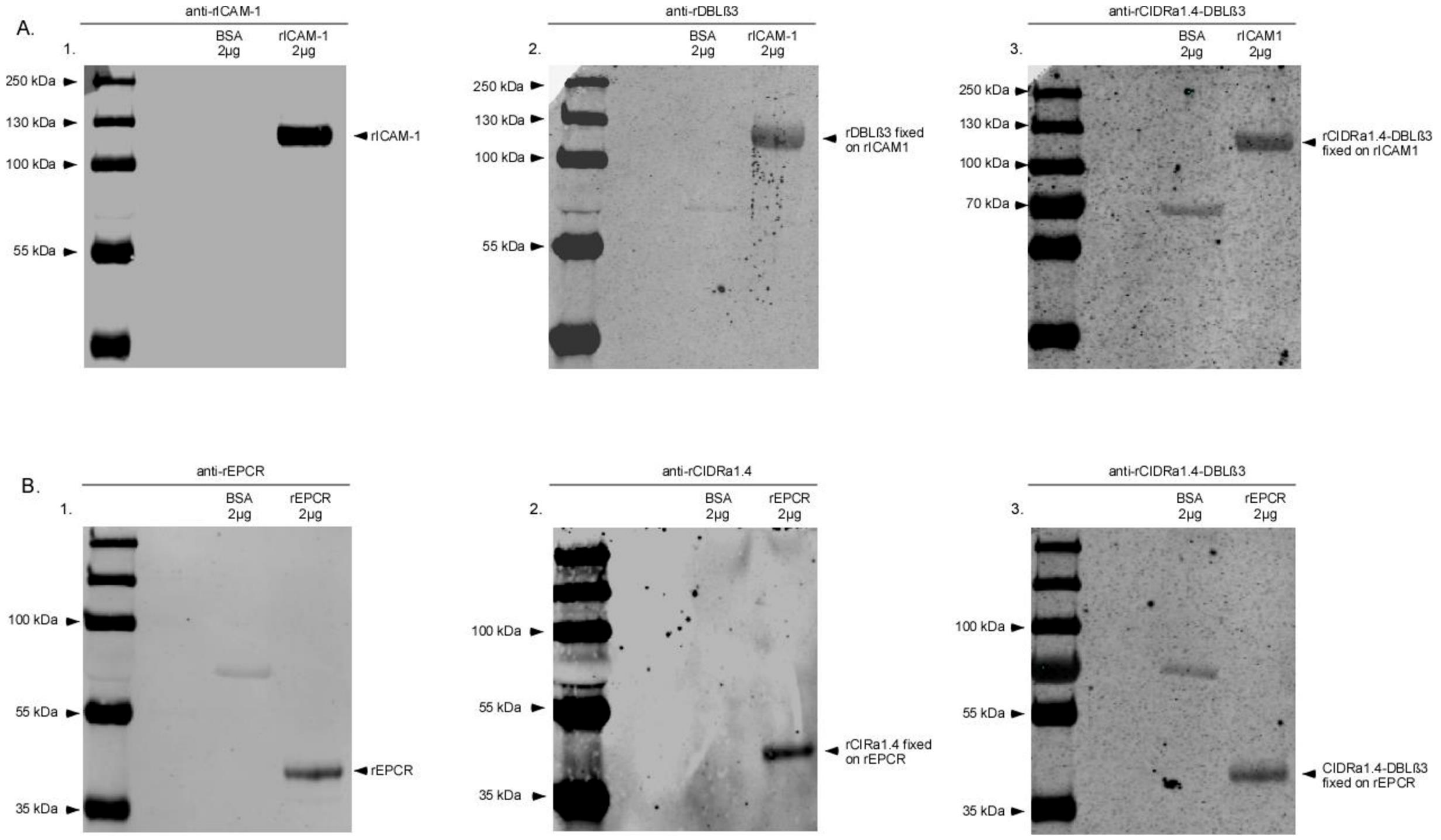

Supplemental figure S2 - Far western blot analysis. **A.1** Western blotting with an antibody against rICAM-1. **A.2** Far western blotting with an antibody against DBLβ3**. A.3** Far western blotting with an antibody against CIDRα1.4-DBLβ3. **B.1** Western blotting with an antibody against rEPCR. **B.2** Far western blotting with an antibody against DBLβ3. **B.3** Far western blotting with an antibody against CIDRα1.4-DBLβ3.
